# Feasibility of an Exercise and CBT Intervention for Treatment of Depression: A Pilot Randomized Controlled Trial

**DOI:** 10.3389/fpsyt.2022.799600

**Published:** 2022-05-04

**Authors:** Jacob D. Meyer, Seana L. Perkins, Cassandra S. Brower, Jeni E. Lansing, Julia A. Slocum, Emily B. K. Thomas, Thomas A. Murray, Duck-chul Lee, Nathaniel G. Wade

**Affiliations:** ^1^Department of Kinesiology, Iowa State University, Ames, IA, United States; ^2^Department of Psychology, Iowa State University, Ames, IA, United States; ^3^Department of Psychological and Brain Sciences, University of Iowa, Iowa City, IA, United States; ^4^Division of Biostatistics, University of Minnesota, Minneapolis, MN, United States

**Keywords:** depression, exercise, cognitive behavioral therapy, physical activity, ActiveCBT, exercise priming

## Abstract

Depression (DEP) is prevalent and current treatments are ineffective for many people. This pilot study's purpose was to assess the feasibility, acceptability, and plausible efficacy of an 8-week intervention employing 30 min of prescribed moderate intensity exercise (“ActiveCBT”) compared to 30 min of usual activities (“CalmCBT”) immediately prior to weekly online CBT sessions. Ten adults with DSM-5-diagnosed current DEP were randomized to groups and completed: an intake assessment, eight weekly CBT sessions, final assessment, and 3-month follow-up. ActiveCBT participants were prescribed 30-min of moderate exercise immediately prior to each standardized 50-min CBT session. CalmCBT participants continued with normal activities for 30 min before therapy. Questionnaires regarding DEP symptom severity (Patient Health Questionnaire-9 [PHQ-9]), between-session effectiveness (Behavioral Activation for Depression Survey [BADS], Automatic Thoughts Questionnaire [ATQ]), in-session effectiveness (Working Alliance Inventory-Short Revised [WAI]), and state anhedonia (Dimension Analog Rating Scale [DARS], Visual Analog Scale [VAS]; assessed 3 times: before Active/Calm condition, after, and after therapy) were completed each week. Therapy fidelity ratings were independently coded *via* a standardized codebook. The Structured Clinical Interview for DSM-5 (SCID) and Hamilton Rating Scale for Depression (HAMD) were used to assess DEP at intake, final, and 3-month follow-up. We found strong feasibility and acceptability (100% adherence, 100% retention at final visit, 74.6% therapy fidelity, and high patient satisfaction ratings). Differences between groups favoring ActiveCBT in anhedonia (DARS, Hedges' *g* = 0.92; VAS, *g* = 3.16), within- (WAI, g = 0.1.10), and between-session effectiveness (ATQ g = −0.65; BADS g = −1.40), suggest plausible efficacy of ActiveCBT for enhancing CBT. DEP rates were reduced in both groups from baseline to final (60% MDD SCID remission) and at follow up (Active: 40%; Calm: 25%). Larger and potentially quicker symptom improvement was found favoring the Active condition to the final visit (HAMD, between-group changes g = −1.33; PHQ-9, g = −0.62), with small differences remaining at follow-up (HAMD, g = −0.45; PHQ-9, g = −0.19). Exercise priming appears acceptable and plausibly efficacious for enhancing mechanisms of CBT and overall outcomes, though the present small sample precludes efficacy determinations. It appears feasible to conduct a randomized controlled trial comparing ActiveCBT to CalmCBT. Future trials evaluating this potentially promising treatment approach and mediating mechanisms are warranted.

## Introduction

Major depressive disorder is prevalent, with the World Health Organization (1) estimating over 322 million people suffer from this condition, leading to it being the largest contributor to non-fatal health loss and suicide across the globe. Current front-line treatments for depression (DEP), such as medication and cognitive behavioral therapy (CBT) are not as effective for many people as they could be (2). Even in patients who respond to treatment, relapse rates are high (3). Increasing the short- and long-term effectiveness of current treatments has the potential to dramatically reduce the immediate and long-term burden of DEP.

Exercise training as a monotherapy can effectively treat DEP (4–7). Previous research shows it is similarly effective to other treatment options (e.g., antidepressant medication), without negative side effects experienced with other treatments [e.g., insomnia, anxiety, cost; (5)]. However, similar to healthy individuals, adherence to regular exercise is low in DEP. In a randomized, controlled trial assessing attendance of adults with DEP (*n* = 310) to an exercise intervention, non-adherence (attending 0 of 12 sessions) and sub-adherence (attending 1-11 of 12 sessions) rates were 40 and 26.9%, respectively (8). Individuals with DEP report substantial barriers to exercise, including lack of motivation, mood, and fatigue, that reduce the likelihood of their regular engagement in this treatment option (9). Though likely difficult to design, the optimal use of exercise to treat DEP would harness the clear and consistent benefits of exercise while addressing adherence concerns.

Due to the immediate short-term psychobiological effects, acute bouts of exercise hold the potential to augment whatever occurs in the window of time after an exercise session. In particular, intentionally sequenced acute exercise could augment the success of psychotherapy if therapy was performed immediately after exercise. Exercise training has been used as an adjunct to therapy previously, but very few studies structure acute exercise before or after therapy when it may affect the quality or potency of the therapy. If exercise were to augment working alliance developed during therapy and CBT session effectiveness (e.g., cognitive changes, behavioral activation), which are strongly linked to DEP response (10–12), the combination could transform treatment success. Szuhany and Otto (13) examined the efficacy of behavioral activation with either exercise training or stretching performed immediately *after therapy* for treatment of DEP. They found both groups experienced symptom improvement over time, with those engaging in more exercise experiencing greater and faster responses. However, if exercise is sequenced immediately *before* therapy (thereby “priming” a person to be ready for therapy), the acute psychological (e.g., lower anhedonia, improved mood, higher self-efficacy) and biological (e.g., increased brain-derived neurotrophic factor [BDNF], endocannabinoids) changes that result from exercise may better prepare patients for engaging in the post-exercise therapy sessions (14–18). Improved mood and anhedonia have been found to last at least 30 min after exercise in adults with DEP, indicating that a standard 50-min post-exercise therapy session could capitalize on these short-term effects (15).

Studies in different psychiatric populations suggest that sequencing exercise before therapy could improve symptoms, though the sample sizes have been small. For example, in a pilot study (*n* = 9), exercise was used immediately prior to exposure therapy for treatment of post-traumatic stress disorder and those in the exercise group experienced greater improvement in symptoms (standardized mean difference = 2.65) and elevated BDNF (standardized mean difference = 1.08) compared to exposure therapy alone (19). Furthermore, in a feasibility trial (*n* = 29) in patients with diabetes and DEP, group exercise prior to therapy was found to be feasible and acceptable by participants. Compared to a therapy-only group, they also found small improvements in exercise enjoyment and areas of behavioral activation, a key mechanism through which CBT may work to alleviate DEP (20–22). Although promising, these results do not provide clear information about the feasibility, acceptability, and efficacy of priming therapy with individualized exercise in the psychotherapeutic treatment of depression. Determining the feasibility, acceptability, and plausible efficacy of this exercise-preceding-therapy sequence is needed to determine the utility of a larger efficacy trial of the exercise priming approach.

Therefore, the purpose of this study was to assess the feasibility and acceptability of an 8-week intervention for DEP prescribing 30 min of moderate intensity exercise (“ActiveCBT”) compared to 30 min of usual activities (“CalmCBT”) immediately prior to weekly manualized CBT sessions. As a pilot feasibility trial, the primary aims of this study were to assess feasibility (recruitment), acceptability (protocol adherence, treatment fidelity, and patient satisfaction), and the plausibility of the effects of exercise priming on CBT [change in state anhedonia across the priming conditions, average session effectiveness measures across treatment, and post-treatment DEP; (23–26)].

## Materials and Methods

### Participants

Participants were recruited via referral from university and community partners, mass emails, and flyers posted locally and in surrounding communities. Inclusion criteria were: willing to comply with all study procedures; compliance with all lifestyle considerations (i.e., refraining from caffeine-containing drinks and using tobacco/nicotine products 4 h prior to sessions, refraining from consuming alcohol and exercising 24 h prior to sessions, and refraining from changing regular medications or treatments during the course of the study); being 18–65 years in age; currently diagnosed with a DSM-5 depressive disorder [i.e., Major Depression Disorder, Persistent Depressive Disorder, Premenstrual Dysphoric Disorder, Other Specified Depressive Disorder; Depressive Disorder Due to Another Medical Condition, Substance/Medication-Induced Depressive Disorder; (27)] assessed *via* the Structured Clinical Interview for DSM-5 [SCID; (28)]; on a stable medication regimen for at least the past 8 weeks and willing to maintain current treatments throughout the study; regular access to a computer or smart phone with internet and teleconference capabilities; currently living in Iowa (due to psychotherapy licensure requirements and COVID-19 related travel restrictions); and access to a private room for virtual study visits. Exclusion criteria were comorbid psychiatric conditions, with the exception of Generalized Anxiety Disorder [GAD—allowed due to high comorbidity; (29)]; activity restrictions that limit the ability to engage in physical activity based on self-reported responses to the Physical Activity Readiness Questionnaire (30); pregnant or planning on becoming pregnant during study enrollment; and previous treatment with CBT in the past 5 years. Participants were also excluded if they answered “Yes” to any of the following conditions: use of illegal drugs, abuse of alcohol or other substances; use of tobacco projects; or taking opioid analgesics, CBD products or cardiovascular medication.

### Procedures

This trial was a randomized, controlled pilot feasibility trial [definitions according to CONSORT guidelines; (26)] evaluating ActiveCBT vs. CalmCBT for enhancing CBT session effectiveness and augmenting overall treatment of DEP. All study procedures were approved by the local institutional review board (Approval #20-105-00). Study procedures consisted of an intake visit, 8 weekly ~2-h sessions of ActiveCBT or CalmCBT, a final visit (intervention spanning 10 weeks), with a 3 month follow up. Data were collected November 2020 through July 2021. Due to the COVID-19 pandemic and associated limitations to in-person research, all study visits were completed electronically using a videoconference software (Cisco Webex, Cisco Systems, San Jose, CA).

For the intake visit, participants joined an electronic meeting and completed informed consent processes. The International Physical Activity Questionnaire-long form [IPAQ; (31)] was then verbally administered to assess current activity levels (see below for further details on questionnaires). Next, participants completed the SCID with a trained doctoral-level Counseling Psychology student interviewer to determine eligibility. The interviewer also completed the Hamilton Rating Scale for Depression [HAM-D; (32)] to assess depressive symptom severity and the Columbia Suicide Severity Rating Scale [C-SSRS; (33)]. Participants that met inclusion/exclusion criteria then completed a second electronic survey consisting of demographic questions, a general health history questionnaire, Anhedonia Visual Analog Scale [VAS; (34)], Dimensional Anhedonia Rating Scale [DARS; (35)], the Patient Health Questionnaire-9 [PHQ-9; (36)], General Anxiety Disorder-7 [GAD-7; (37)], and Short-Form Health History [SF-36; (38)]. Participants were then randomized to condition *via* selection of a sequentially numbered set of opaque envelopes. Randomization sequence had been performed prior to study initiation and all staff members were blind to order. All participants were then provided with a commercial activity monitor (Fitbit Alta HR, Fitbit Inc, San Francisco, CA) to wear during the duration of the study. Lastly, all therapy sessions were scheduled, with the first beginning within 1 week of the intake visit.

During the 8-week intervention period, participants engaged in weekly online individual CBT sessions. Prior to each therapy session, participants completed questionnaires before and immediately after a 30-min Active or Calm condition. The first set of questionnaires included Anhedonia VAS, DARS, PHQ-9, Behavioral Activation for Depression Scale [BADS; (39)], and Automatic Thoughts Questionnaire [ATQ; (40, 41)]. The IPAQ was also verbally administered. Individuals randomized into the Active condition were then instructed to exercise at a “Somewhat Hard” intensity or Rating of Perceived Exertion (RPE) equal to 13 (moderate) consistently, with a built-in 3-min warm-up and cool down. Participants self-selected the exercise location, but were asked to exercise in the same location each time to minimize inter-session variability. Individuals randomized to CalmCBT were asked to continue with normal daily activities prior to their CBT session. Upon completion of the Active or Calm condition (immediately before therapy), participants completed a second set of questionnaires including the Anhedonia VAS and DARS. Participants then began a 50-min CBT session with a trained therapist (advanced counseling psychology doctoral student supervised by NW), using *A therapist's guide to brief cognitive behavioral therapy* (42). To monitor suicidality during each session, the therapist administered the CSSRS. During Visit 4 (mid-point assessment), the HAM-D was also completed by the therapist in-session to assess depressive symptom severity. To ensure standardization of the sessions within and between participants, recordings (audio and video) of the sessions were collected and transcribed using Webex. In line with the CBT manual (42), overarching goals of treatment included: behavioral activation and engagement; identifying automatic thought patterns; observing connections between thoughts, emotions, situations, and behaviors; and identifying cognitive distortions. Immediately after each therapy session, participants completed a third set of questionnaires including the Anhedonia VAS, DARS, Working Alliance Inventory-Short Revised [WAI-SR; (43)], and Session Effectiveness Questionnaire (SEQ).

Participants completed a final visit within 1 week of the last CBT session and a follow up visit 3 months after the final visit. Both visits were identical to the intake visit with the addition of the Subjective Treatment Satisfaction Survey [STSS, administered during both visits (44)] and the Qualitative Change Interview [final visit only; (45)]. In each visit, the HAM-D and SCID were completed by the same-trained interviewer who had completed the intake visit who was blind to group assignment.

### Feasibility Measures

Efficacy of recruitment methods was tracked by having each participant respond to a single item question on the screening survey, inquiring about how they heard about the study. Response choices were specific to each recruitment method (e.g., email, flier, word-of-mouth) and community partner. Data regarding eligibility were also recorded during the screening process.

### Acceptability Measures

#### Protocol Adherence

Adherence to the protocol was measured by recording of intervention scheduling processes and participant attendance to study visits, allowing for quantification of adherence rates (i.e., percent of study visits within an acceptable window) and retention (i.e., percent who completed all study visits). Protocol adherence was operationally defined on a session level (i.e., weekly CBT session) with each CBT session occurring 7 ± 2 days after each preceding visit. Retention was operationally defined as the percentage of participants that completed all 10 study visits (not including the 3-month follow-up).

#### Condition Adherence

Condition adherence (i.e., pre-therapy 30-min Active and Calm conditions) was evaluated using data collected from the Fitbit monitor. Minute by minute data from the intervention period was recorded and exported using Fitabase (Fitabase, Small Steps Labs LLC, San Diego, CA), and each 30-min condition period was isolated to calculate heart rate and total step count.

#### Therapy Fidelity and Safety

Fidelity was evaluated by transcribing video recordings of CBT sessions. A randomly selected 25% of all session transcripts were retrospectively coded by one of the other therapists who did not perform the therapy session. Criteria for coding was selected based on a pre-defined fidelity checklist with 5 items for each session rated 0–2, with 0 indicating “not covered at all,” 1 indicating “reference to topic and quick review,” and 2 indicating “topic was adequately covered.” Items were summed for an overall session fidelity rating score of 0–10, with higher scores indicating greater CBT congruency and alignment with weekly session goals outlined in the CBT manual (42). Participant safety was also monitored by therapists at each visit using the C-SSRS. Data regarding suicide ideation, suicide behavior, completed suicide and other adverse events were used to evaluate the effectiveness of the study safety plan.

#### Patient Satisfaction

Patient satisfaction was assessed *via* the STSS, a self-reported quantitative treatment satisfaction questionnaire, and a qualitative semi-structured interview administered during the final study visit. The STSS was used to quantitatively assess acceptability by having participants respond to 6 items (Likert scale responses) regarding their perception of the effectiveness and satisfaction of the treatment (44). A total score was calculated, ranging from 0 to 46, with higher scores indicating greater satisfaction. In addition, a semi-structured interview based on the Change Interview for Psychotherapy was administered (45). Interviews were all recorded, transcribed, quality checked, and coded to evaluate satisfaction of overall treatment and group allocation (detailed below).

### Plausibility of Efficacy Measures

#### State Anhedonia

State anhedonia was assessed using both an anhedonia VAS and the DARS. Using the VAS, participants responded to the prompt “Drag the slider to rate how you are feeling right now,” on a sliding scale from 0 (“No pleasure at all”) to 100 (“Extreme pleasure”). The DARS was also administered at each timepoint. The DARS is a 17-item self-report survey that assesses state anhedonia across four domains: hobbies, food/drink, social activities, and sensory experiences. Subjects listed two activities within each domain that they consider their favorite and then respond to questions regarding their current interest in and enjoyment of those activities on a 5-point Likert scale from “Not at all” to “Very much.” Participants were asked to respond to how they are feeling “RIGHT NOW” (35). Favorite items were recorded at the intake visit and then those two favorite items were used as the prompt for each subsequent completion of the instrument. Higher scores represent less anhedonia.

#### Session Effectiveness

The SEQ, WAI-SR, BADS, and ATQ were administered to assess within (i.e., SEQ, WAI-SR) and between (i.e., BADS, ATQ) session effectiveness. The full SEQ includes 21 items in a 7-point bipolar adjective format that query how the client feels about each CBT session (46). The subscales of session depth, smoothness, and the first, single item session evaluation question (i.e., “*This session was…”* with scale anchors of “bad” to “good”) were calculated to assess within-session effectiveness. The WAI-SR is a 12-item survey over the experience in the therapy session and with the therapist. Each item is assessed using a rating scale from 1 (seldom) to 5 (always), to calculate a total score and subscales of tasks, goals, and therapist-client bond. Higher total scores and subscales indicate greater therapeutic alliance. The BADS is a scale designed to measure behavioral activation in the preceding week. It consists of 25 questions that inquire about Activation, Avoidance/Rumination, Work/School Impairment, and Social Impairment (sub-scaled), with subscales summed for a total score with higher total scores reflecting higher behavioral activation (47). Lastly, the ATQ is a 30-item instrument that measures the frequency of automatic negative statements about the self. Each item is rated on the frequency of an occurrence from 0 (“not at all”) to 5 (“all the time”), with higher total scores reflecting greater frequency of automatic negative self-statements.

#### Depression

The SCID, HAM-D and PHQ-9 were used to assess DEP. The SCID was administered to determine DEP diagnosis at intake, the final visit and the follow up visit. The SCID is a semi-structured interview guide for making diagnoses according to the diagnostic criteria published in the American Psychiatric Association's Diagnostic and Statistical Manual for Mental Disorders (DSM-5; 2013). The HAM-D was assessed during intake, mid-treatment (i.e., CBT session 4), the final visit and the 3-month follow up visit. It is a 17-item clinician-completed questionnaire used to rate depressive symptom severity by probing mood, feelings of guilt, suicidal ideation, insomnia, agitation or retardation, anxiety, weight loss, and somatic symptoms (48). Higher scores reflect greater symptom severity, categorized as Normal (0–7), Mild Depression (8–13), Moderate Depression (14–18), Severe Depression (19–22), and Very Severe Depression (>23). Interviewers for the SCID and HAM-D were conducted by Counseling Psychology doctoral students trained in the use and scoring of the instruments by NW, a licensed psychologist with over 10 years of supervision experience. Interviewers were blind to treatment assignment. Lastly, the PHQ-9 was used to evaluate weekly changes in self-reported depressive symptoms. This is a 9-question self-report instrument used to assess presence and severity of depressive symptoms, with scores categorized as minimal (0–4), mild (5–9), moderate (10–14), moderately severe (15–19), and severe (20–27) depression (36).

### Statistical Analysis

Descriptive statistics were calculated to describe participant demographic characteristics, feasibility, acceptability, and plausibility of efficacy of exercise priming for enhancing CBT. Participant characteristics were described by median, minimum, and maximum values for continuous variables, and total number of participants and proportions for categorical variables (see Table 1).

**Table 1:** Participant characteristics.

	**Active (*n* = 5)** **Median [min, max]** **or *n* (%)**	**Calm (*n* = 5)** **Median [min, max]** **or *n* (%)**
**Demographics**		
Age	46 [35, 59]	44 [19, 51]
Sex (female)	4 (80)	5 [100]
BMI	26 [22, 38]	34 [19, 44]
Race (white)	5 (100)	4 (80)
Education (graduate degree)	2 (40)	3 (60)
Employment (full time)	5 (100)	4 (80)
Income ($50–75K)	3 (60)	2 (40)
**Psychiatric Diagnoses from SCID**		
Lifetime Major DD (*n* = 9) or Other Specified DD (*n* = 1)	5 (100)	5 (100)
Current Major DD	3 (60)	4 (80)
Current Persistent DD	2 (40)	2 (40)
Current Other Specified DD	2 (40)	1 (20)
Comorbid Premenstrual Dysphoric Disorder	1 (20)	1 (20)
Comorbid Generalized Anxiety Disorder	1 (20)	1 (20)
**Baseline symptom severity**		
Severity of DEP symptoms (HAM-D total)	11 [3, 16)]	11 [7, 12]
Self-reported DEP symptoms (PHQ-9 total)	17 [6, 19]	14 [8, 16]
Current DEP medication use (SSRI and NDRI)	2 (40)	2 (40)
Anxiety severity (GAD-7)	17 [8, 19]	5 [3, 14]
Physical health quality of life (SF-36)	53 [50, 66]	55 [31, 68]
Mental health quality of life (SF-36)	21 [5, 34]	22 [11, 39]
**Baseline activity level**		
Steps/day	5,731 [1,957, 10,875]	4,424 [2,806, 6,780]
MVPA (min/day)	12 [2, 19]	13 [2, 53]
Sedentary time (h/day)	16.2 [14.5, 18.4]	17.3 [11.5, 18.7]

*Four participants were diagnosed with 2 types of current depressive disorders; Quality of life data from SF-36 are physical and mental health composite scores; Step, MVPA, and sedentary data from activPAL monitor collected between the intake visit and first therapy session. min, minimum value; max, maximum value; BMI, body mass index; DD, depressive disorder; HAM-D Total, total score from Hamilton Rating Scale for DEP; SSRI, selective serotonin reuptake inhibitor; NDRI, norepinephrine and dopamine reuptake inhibitors; GAD Total, total score from Generalized Anxiety Disorder-7 Scale*.

**Figure 1:**
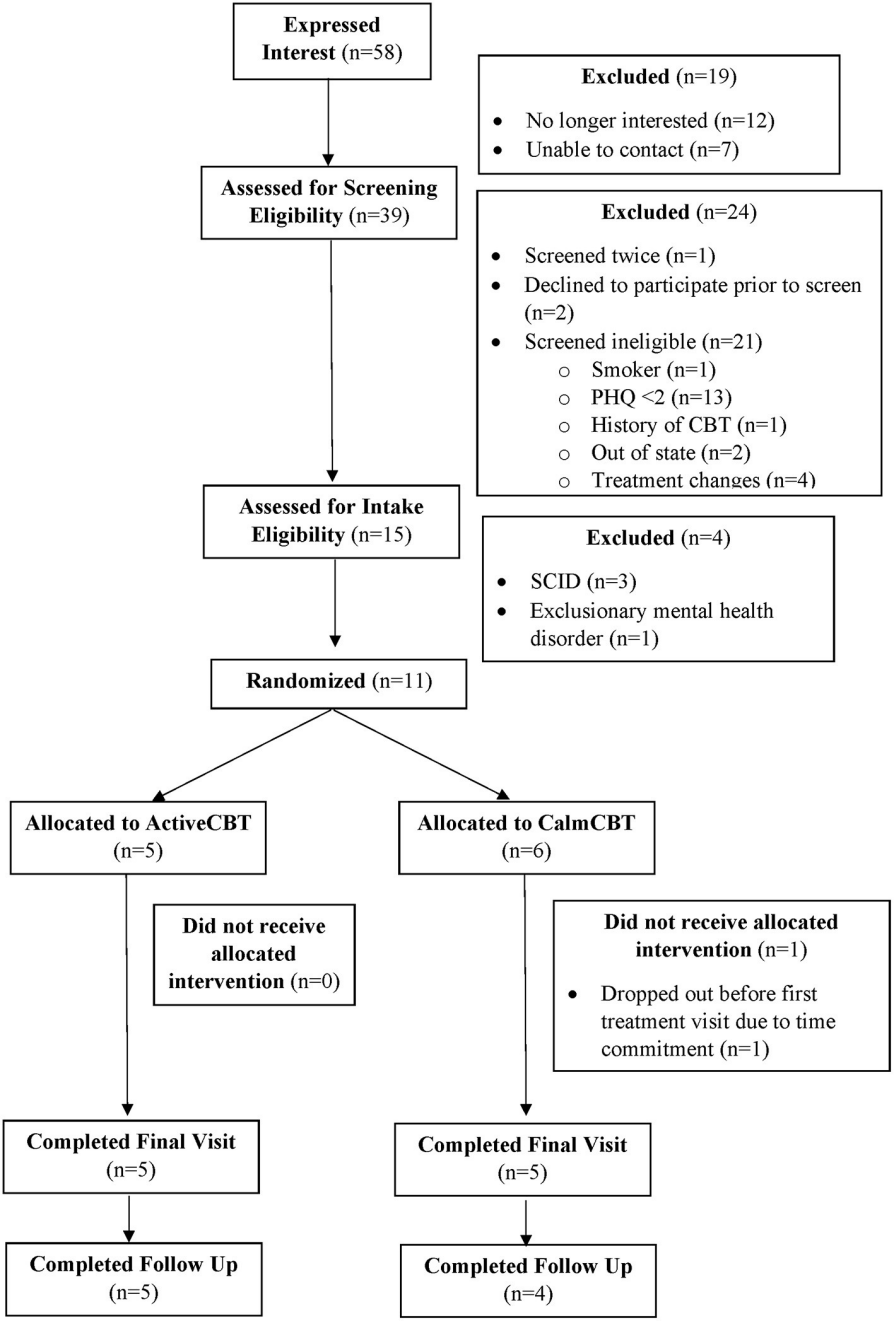
CONSORT diagram. PHQ, Patient Health Questionnaire; CBT, Cognitive Behavioral Therapy; SCID, Structured Clinical Interview for Depression.

Recruitment rates, referrals from community partners, refusal rates, screening failures, and participant flow are illustrated in a CONSORT diagram (see Figure 1).

To assess protocol adherence, percentages of adherence (i.e., CBT session occurring 7 ± 2 days after each preceding visit) and retention (i.e., participants that completed all 10 study visits) were calculated. Condition adherence was examined by calculating heart rate variables (i.e., resting, minimum, maximum, and average) and total steps from the Fitbit during the 30-min condition (i.e., Active or Calm) period. Therapy fidelity was assessed by describing mean ratings of session transcripts and participant safety was evaluated using total scores of suicide ideation and behavior, completed suicide, and other adverse events. Patient satisfaction was explored with median, minimums and maximum scores of the STSS and final themes from the qualitative data analysis.

The qualitative data were assessed using a thematic analysis to understand perceived effectiveness and overall satisfaction, using processes recommended by Miles et al. (61) and employed previously (49). Each interview was recorded with an automatic transcription created using Webex and manually cross-checked for errors. Three coders (JL, JS, & SP) were responsible for jointly creating a deductive codebook to guide initial coding, before two coders (JL & JS) separately identified codes for each conversation. All coders then met to compare and finalize first-cycle coding. Data units were indexed and charted, and subsequently the coders met to compare coding and establish final thematic categories and associated definitions (i.e., second-cycle coding). A thematic matrix was then created based on final themes, definitions, and exemplary participant quotes.

Means, SDs, and effect sizes [Hedges' *g* was used including the small sample size correction to account for small group sizes; (50)] were used to explore changes in anhedonia VAS and DARS total (i.e., pre-exercise to post-exercise and pre-exercise to post-therapy, within and between group for each questionnaire), with estimates over 0.2, 0.5, and 0.8 being reported as small, medium, and large effects, respectively. Plausibility of differences in session effectiveness was examined by comparing means, SDs, and effect sizes between groups for SEQ total, BADS total, and ATQ total. Changes in DEP outcomes were computed and descriptively compared for the SCID (i.e., total participants meeting MDD criteria at baseline, during the final assessment, and follow up visit by group) and the HAM-D (i.e., final and follow up total score minus baseline total score). Weekly changes in depressive symptoms from the PHQ-9 were also examined by group. Overall missingness was extremely low with 0.38% of values missing. Missing data was accounted for using mean imputation for DARS anhedonia and session effectiveness measures, and a last observation carried forward approach was used for depression outcomes. Finally, on the VAS, scores of 50 were imputed if the VAS slider remained over the midline (i.e., at 50) when no raw value was recorded (i.e., REDCap indicated “no response” because the participant did not move the slider). Graphical data depict raw values while tabular data and data presented in-text represent estimated means, standard deviations, and associated effect sizes.

## Results

### Feasibility

Employed recruitment strategies yielded 58 interested potential participants in 5 months, resulting in an average recruitment rate of ~12 interested potential participants each month. The primary mode of recruitment for those who completed the screening survey was through referrals from community partners (*n* = 16) supplemented by an email to select campus community members (*n* = 10), lab social media or websites (*n* = 8), and flyers (*n* = 5), demonstrating that colleagues, community partners, and the campus community were effective sources of recruitment. Refusal rates and reasons for ineligibility are presented in Figure 1. Seventy-three percent of those screened as eligible were also eligible after the intake visit, suggesting these are feasible and suitable screening and inclusion criteria. Eleven participants enrolled in the study, with one participant (randomized to CalmCBT) withdrawing before the first treatment due to the time commitment. Participant characteristics of the 10 participants who received the intervention are displayed in Table 1.

### Acceptability Measures

#### Protocol Adherence

All 10 participants who attended their first CBT session were retained through the final visit (i.e., 100% retention rate for those who came to their first session; 91% retention rate for those randomized). One participant was lost to follow-up at 3 months (i.e., 90% retention rate from baseline to 3-month follow-up). Out of 90 total appointments (i.e., 80 CBT sessions and 10 final assessments), 86 occurred 7 ± 2 days from the previous week's appointment (96%). Eleven total appointments were rescheduled (planned or unplanned) due to the visit occurring on a holiday (*n* = 4), participant traveling out of state (*n* = 1), or a scheduling conflict (*n* = 6). Out of the nine 3-month follow-up assessments, four occurred during the projected 3-month week (44%), four occurred 1 week later than the projected date, and one occurred 1 month after the projected date.

#### Condition Adherence

Due to COVID-modified procedures, exercise occurred outside of the laboratory, wherever participants were able to complete it. During the Active condition, three participants selected walking (2 outdoor, 1 indoor) as the mode of exercise. Due to weather and for convenience, one participant chose to cycle on a stationary bike indoors and one participant completed a moderate intensity workout video (i.e., intervals of aerobic bodyweight exercises) during the condition time. Individuals in the Calm condition reported resting, continuing with seated work, reading, and watching television. Averages of Fitbit data from the 30-min conditions are shown in Table 2. Briefly, participants who walked and biked or used a workout video accumulated over 3,000 and 1,000 more steps (respectively by mode, with fewer steps recorded by the wrist-worn Fitbit monitor during biking and video workouts) and had higher average heart rates than those in the Calm condition, suggesting adherence to assigned conditions.

**Table 2:** Condition adherence.

**Condition Variable**	**ActiveCBT (*n* = 5) Median [min, max]**	**CalmCBT (*n* = 5)** **Median [min, max]**
Resting Heart Rate (bpm)	76 [63, 81]	77 [55, 79]
Minimum Heart Rate (bpm)	83 [67, 91]	74 [53, 82]
Maximum Heart Rate (bpm)	123 [115, 147]	95 [70, 113]
Average Heart Rate (bpm)	107 [95, 118]	83 [61, 90]
Total Steps	3,128 [954, 3,864]	64 [23, 176]

*Data are median (minimum-maximum) values during the eight, 30-min Active or Calm conditions (immediately before therapy), collected from the Fitbit monitor. Resting Heart Rate is an average of the participants resting heart rate for the week (i.e., 7 days) prior to the CBT session. All heart rate values are beats per minute (bpm)*.

#### Therapy Fidelity and Safety

Three Counseling Psychology doctoral students were successfully trained in the delivery of standardized CBT and DEP outcome assessments (i.e., SCID, HAM-D, and CSSRS) by NW in 2 months. Two of the therapists served as fidelity coders and each coded 10 CBT sessions. The mean session fidelity rating was 7.45 ± 1.8. Participant safety was successfully monitored at each visit. Suicide ideation and behavior were low and decreased from baseline (only one non-zero score which was 2) to the final visit and follow-up (all 0 scores), with no attempted or completed suicides or other adverse events.

#### Patient Satisfaction

At the end of treatment, participants in both groups reported high overall treatment satisfaction on the STSS [median [minimum, maximum] values; Active: 46 (44, 48), Calm 40 (32, 47)]. High satisfaction and perceived effectiveness were also reported by participants in the qualitative analysis. Final themes of satisfaction were *Satisfaction with Overall Experience & Study Logistics* and *Satisfaction with Therapy*. Themes of perceived effectiveness were *Effectiveness of Active Condition for Physical Health* (Active *n* = 3), *Effectiveness of Active Condition for Mood Enhancement* (Active *n* = 5), and *Effectiveness of Both Conditions for Therapy Preparation* (Active *n* = 3; Calm *n* = 4). Table 3 displays a thematic matrix of these results. Participants also reported that reducing the number of total surveys per visit, wearing the activPAL activity monitor less often (e.g., no mid-point), and extending the total CBT session number (e.g., 10 weeks) could further improve the experience.

**Table 3:** Thematic matrix of qualitative analysis.

**Theme**	**Definition**	**Example of Participant Quote**
**Satisfaction**		
Overall experience and study logistics	Participants expressed high satisfaction with the overall research study, including overall satisfaction, interactions with team members, acceptable weekly session length, and convenient format (e.g., flexible scheduling, electronic surveys, teletherapy).	*If somebody asked me, should I do this? I would say absolutely! I felt like it was very productive and a good use of my time*. *Everybody down to the youngest undergraduate [student] were professional and friendly*. *I was pleasantly surprised and pleased with how much work we were able to do in such a short period of time, and how impactful it was*.
Therapy	Participants expressed high satisfaction with therapy, including the therapeutic relationship (e.g., exceptional therapists with objective perspectives, nonjudgmental, accepting), perceived effectiveness of focused CBT approach, gained skill set (e.g., fact-checking, addressing distorted perceptions, increased resilience), and changed negative perception of therapy efficacy.	*I've been to multiple counselors throughout my lifetime, and I truly feel that this has been the best experience, and the best one that has equipped me with tools*. *I have a set of questions I can strategically ask myself in order to make better decisions for my self-care [and] my wellbeing*. *I was able to adopt that kind of mindset, questioning objectively some of what I was doing, and so our conversations allowed me to develop a better self-awareness to make better decision-making skills or to make better decisions*.
**Perceived Effectiveness**		
Active condition for physical health	Participants expressed that the active condition was a positive exposure to exercise, reporting it was helpful in recognizing the immediate-felt benefits of exercise, learning the transferability of physical activity into daily life, building of an exercise routine, and improving sleep.	*I think it was helpful in getting me to do regular exercise… most days…I ended up doing about half an hour on the bike. So, it was a good pattern setter*. *I think one of the next steps I need to add to the toolbox of the skills that I've gained is trying to fit in some exercise, because I think that will only help myself more in the feeling-good spectrum*.
Active condition for mood enhancement	Participants expressed that the active condition was helpful for immediately improving mood, reported as increased energy, confidence, sense of self-efficacy, active engagement in therapy, and overall positive affect.	*You feel confident after exercising and I think that's a great mind space to be in when you go into therapy and start talking about difficult, tough things. It's good to carry those endorphins, that confidence, and that sense of wellbeing into the therapy session…I think going into it with that sort of extra energy was positive in its impact on the effectiveness of the therapy*. *[Exercise] is like fertilizer. You can plant stuff in the garden in your backyard and it might grow just fine. Right? But if you have that little extra fertilizer that you put into the ground beforehand, then the ground has been prepared, and so I feel like the exercise was maybe like preparing me to go into therapy and be willing to be challenged*. *I felt very good, and I also noticed as I was doing the questionnaires…I always just felt more energized, like, yeah, of course I would eat all of this! Of course, I would go [favorite activity]! Of course I would [favorite social activity]!…Of course, it makes all the sense in the world, like, your endorphins get up, your heart rate and everything is [up], and all of that stuff is happening in your body and you go into therapy and that transfers over*.
Both conditions for therapy preparation	Participants in both conditions expressed the condition was helpful in preparing for therapy sessions by providing an opportunity to mentally detach from outside stressors (e.g., work), reflect on previous CBT sessions and homework assignments, and plan for and prioritize the issues to bring up in the upcoming CBT session.	*It allowed me to pre-digest, or maybe post-digest some of the stuff that had happened the week before…and to process through what we had talked about before so that when I came into therapy…I just felt much more, like, ready and, like, present…* *Having that gap in between work and starting the session I felt was helpful because it gave me a chance to get out of the workspace and kind of just settle before I got into actually dealing with therapy…I did feel like having a time in between life and starting gave me a chance to become calmer and to also focus on or let go of the crazy and just focus on the things we're [going to] talk about*.

### Preliminary Efficacy

Estimated means and standard deviations for measures of anhedonia, session effectiveness and depression are presented in Table 4 based upon median, minimum, and maximum values as recommended in Hozo et al. (51). Hedges' g values are also included based upon the estimated mean values and estimated standard deviations after correction for small sample sizes (50).

**Table 4:** Estimated mean and standard deviation values for changes in anhedonia, session effectiveness, and overall depression outcomes across the intervention and 3-month follow-up.

	**ActiveCBT (*****n*** **=** **5)**		**CalmCBT (*****n*** **=** **5)**		
	**Mean**	**SD**	**Mean**	**SD**	**Hedges' g**
**Anhedonia**					
DARS Condition Change	5.83	5.65	1.3	2.57	0.92
DARS Therapy Change	11.2	6.88	5.1	4.15	1.44
VAS Condition Change	8.16	2.52	1.06	1.37	3.16
VAS Therapy Change	10.5	4.74	8.09	3.30	0.53
**Session Effectiveness**					
WAI-SR	57.8	1.74	53.8	4.32	1.10
ATQ	49.4	5.17	54.0	7.5	−0.65
SEQ Depth	6.43	0.27	5.78	0.57	−1.34
SEQ Smoothness	5.33	1.03	5.36	0.49	−0.04
SEQ Positivity	6.06	0.48	5.56	0.50	0.91
SEQ Arousal	4.73	0.61	3.59	0.31	2.12
SEQ Overall	6.88	0.07	6.53	0.42	1.04
BADS Total	103.5	13.4	86.8	7.15	1.40
**Depression**					
PHQ-9 Change at Final	−9.5	4.05	−7.3	2.26	−0.62
PHQ-9 Change at Follow Up	−5.8	4.96	−4.75	4.17	−0.19
HAMD Change at Final	−8.8	2.69	−5.0	2.38	−1.33
HAMD Change at Follow Up	−2.8	2.03	−1.25	3.76	−0.45

*Data represent estimated mean and standard deviations from (51) using median, minimum and maximum values. Condition and therapy changes in DARS and VAS represent the estimated group mean and SD for values averaged across all 8 sessions per participant. Hedges' g represents the standardized mean difference after correction for small sample sizes. DARS, Dimensional Anhedonia Rating Scale; VAS, depression Visual Analog Scale; WAI-SR, Working Alliance Inventory-Short Revised; ATQ, Automatic Thoughts Questionnaire-30; SEQ, Session Evaluation Questionnaire; PHQ-9, Patient Health Questionnaire-9; HAMD, Hamilton Rating Scale for Depression-17*.

#### Anhedonia

**Figure 2:**
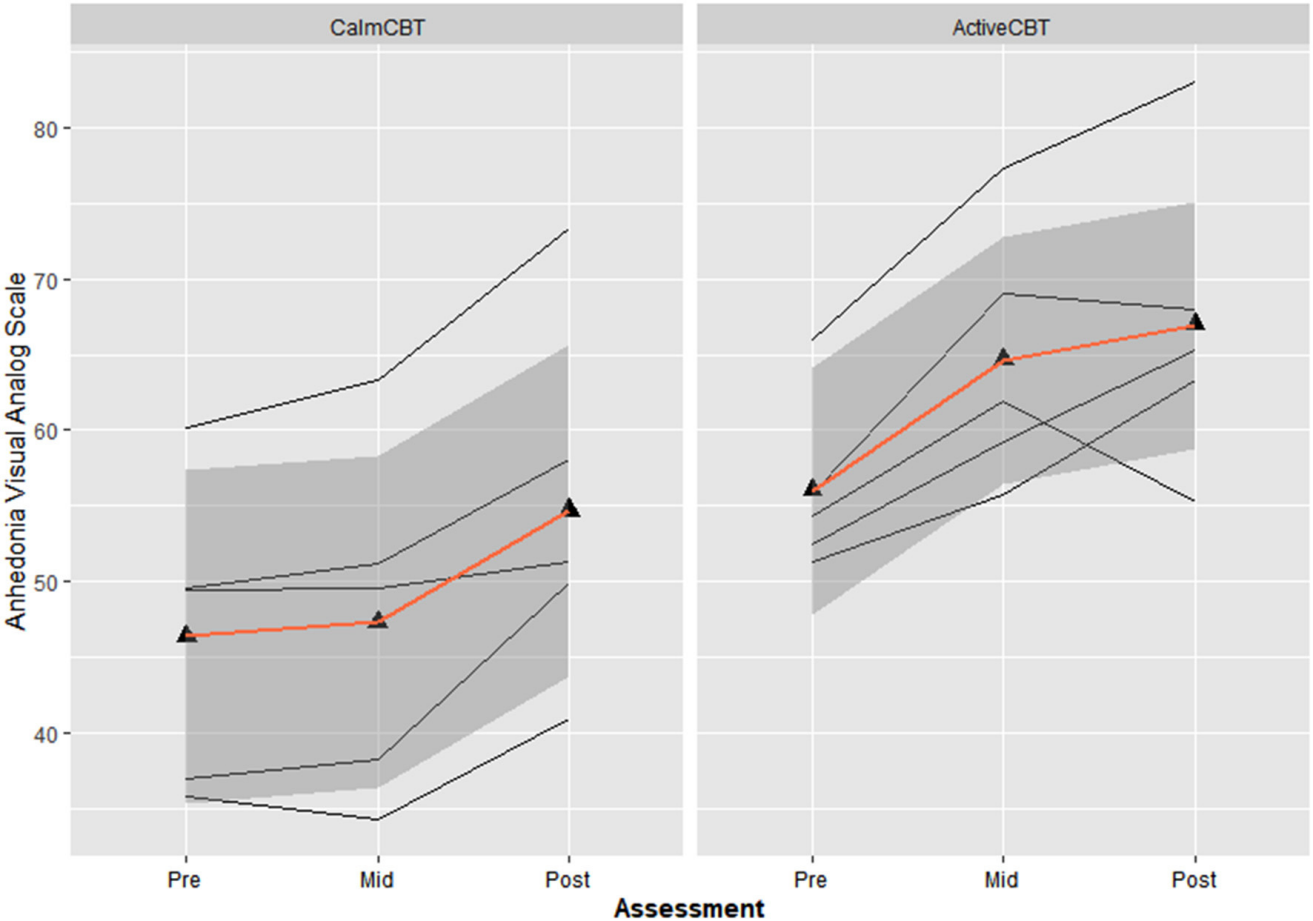
Changes in anhedonia visual analog scale (VAS) within sessions by group. Average changes across all 8 visits in Anhedonia VAS ratings immediately before the Active or Calm condition (i.e., Pre-Condition), immediately after the condition (i.e., Post-Condition) and immediately after the 50-min CBT session (i.e., Post-Therapy). Higher Anhedonia VAS ratings indicate lower levels of anhedonia.

**Figure 3:**
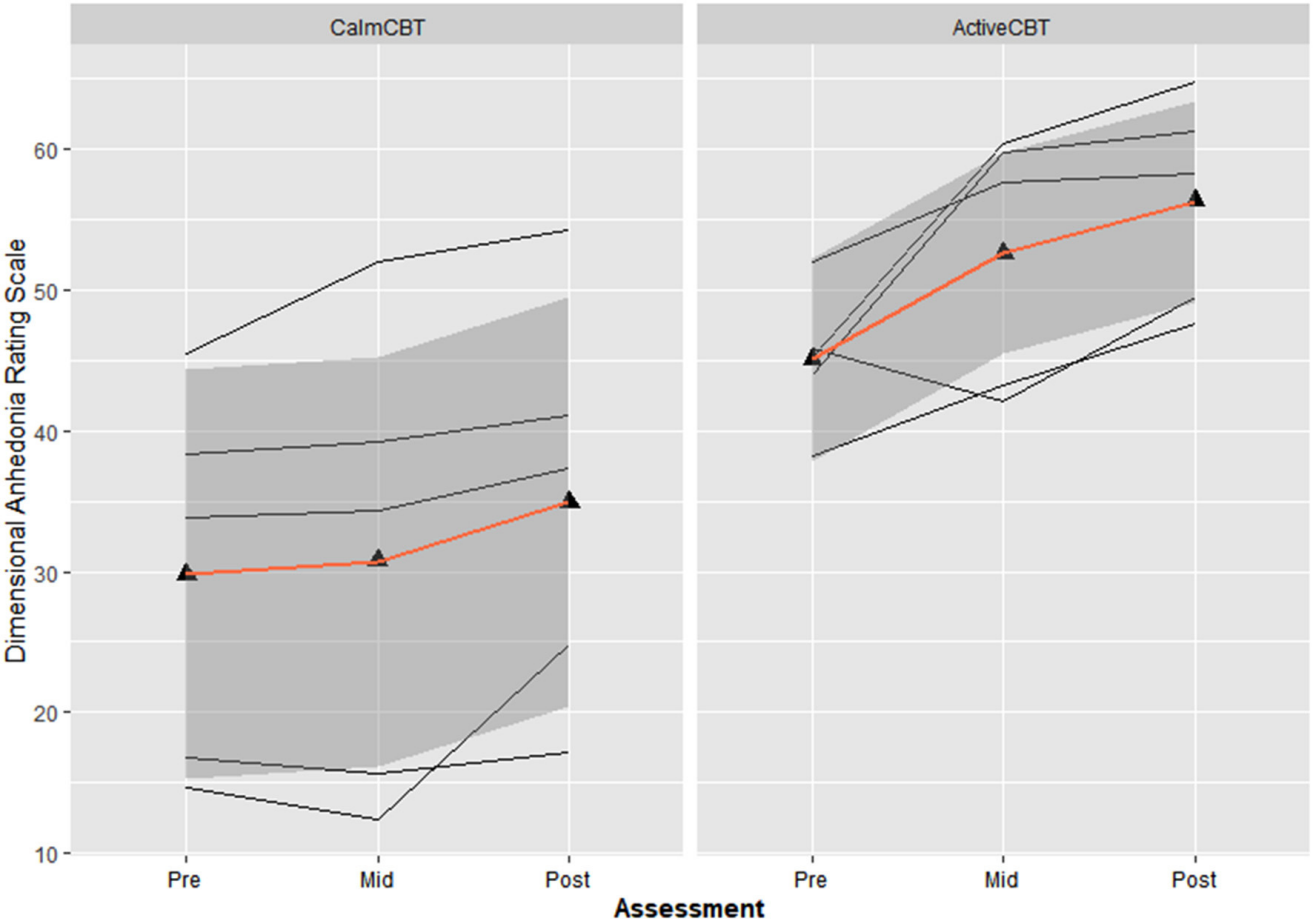
Changes in dimensional anhedonia rating scale (DARS) within sessions by group. Average changes across all 8 visits in the total score of the DARS immediately before the Active or Calm condition (i.e., Pre-Condition), immediately after (i.e., Post-Condition) and immediately after the 50-min CBT session (i.e., Post-Therapy). Higher scores indicate lower levels of anhedonia.

Both measures of anhedonia appeared to improve from pre-condition to post-condition to post-therapy in the Active condition. Session averages at each time point for the VAS and DARS are presented in Figures 2, 3, respectively. Large differences in changes in pleasure were observed between groups from pre to post condition on both the VAS (*g* = 3.1) and DARS (*g* = 0.92), with less anhedonia reported by those in the Active group.

#### Session Effectiveness

**Figure 4:**
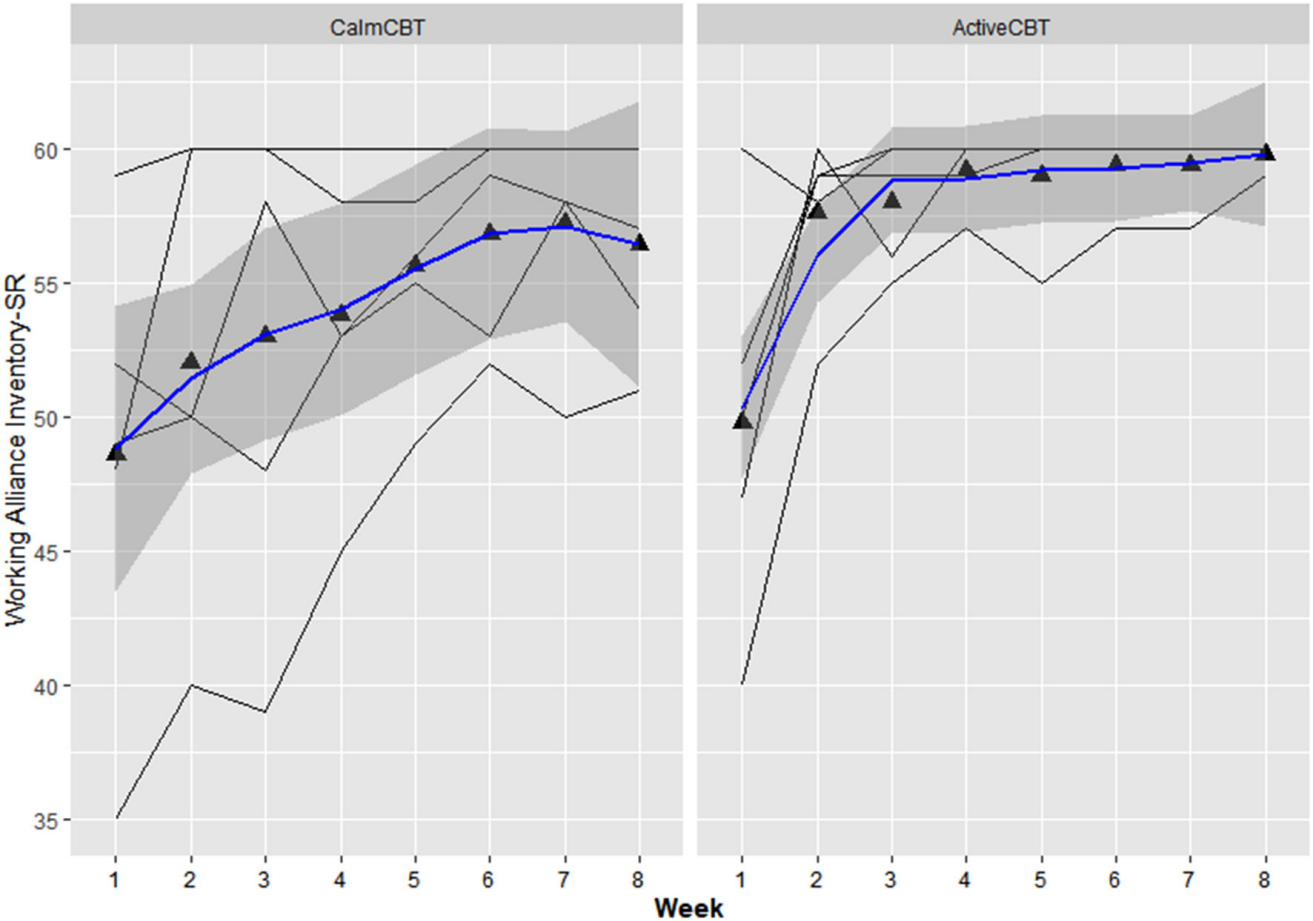
Working alliance inventory short revised (WAI-SR) by group over time. Data are total scores of the Working Alliance Inventory (range 15–60) administered at the end of each CBT session. High scores indicate a stronger therapeutic alliance.

**Figure 5:**
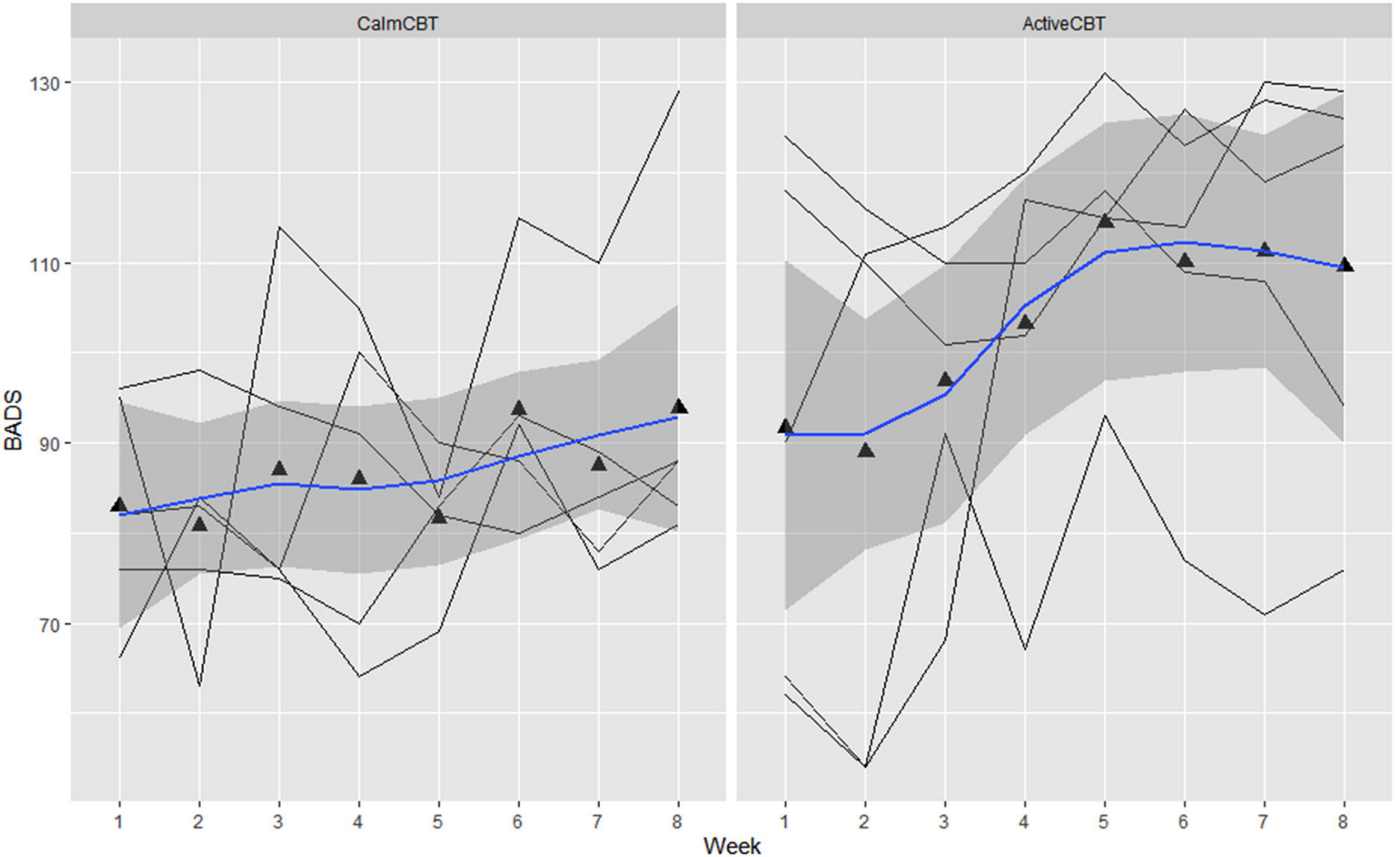
Behavioral activation for depression scale (BADS) by group across time. Data are total scores of the BADS administered at the beginning of each CBT session visit (i.e., before the Active/Calm condition). High scores indicate greater levels of reported behavioral activation.

**Figure 6:**
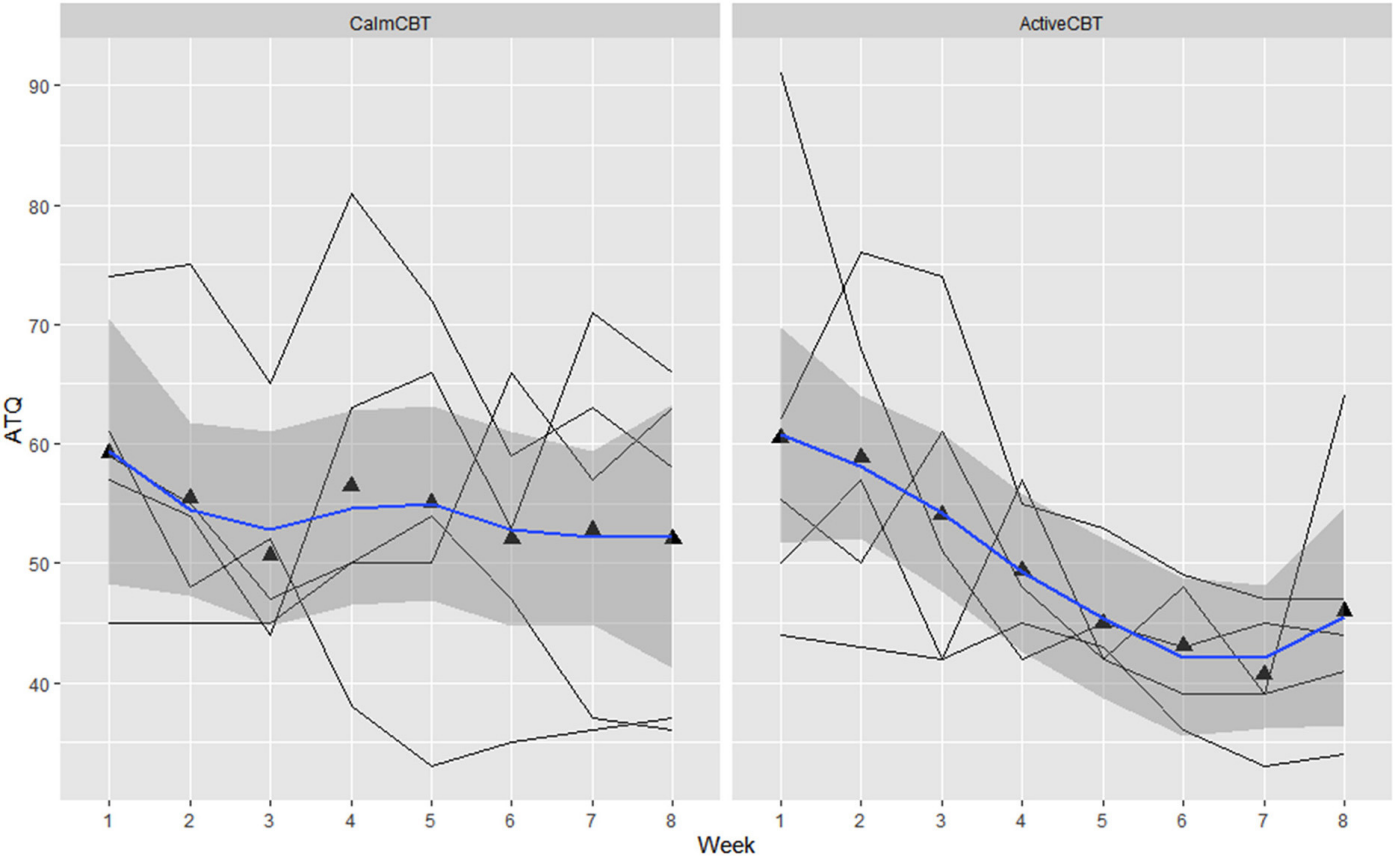
Changes in automatic thoughts questionnaire (ATQ) by group over time. Data are total scores of the ATQ administered at the beginning of each CBT session visit (i.e., before the Active/Calm condition). Lower total scores indicate lower frequency of negative thoughts.

Within-session effectiveness appeared higher for ActiveCBT participants on the SEQ, shown by differences in estimated means across all 8 visits of session depth (*g* = 1.34), depth (*g* = 0.91) and arousal (*g* = 2.12), and response on the overall session effectiveness question (*g* = 1.04), though there appeared limited difference. Estimated means of therapeutic alliance (i.e., WAI-SR Total) was also higher for (*g* = 0.75), with what appeared to be quicker gains in alliance observed in the Active group, shown in Figure 4. Higher behavioral activation was also reported over time (Figure 5), with higher behavioral activation estimated in the ActiveCBT group (*g* = 1.05). Negative automatic thoughts also appeared to be reduced over time in both groups (Figure 6), with somewhat less frequent negative thoughts estimated in the ActiveCBT group (*g* = −0.65).

#### Depression

**Figure 7:**
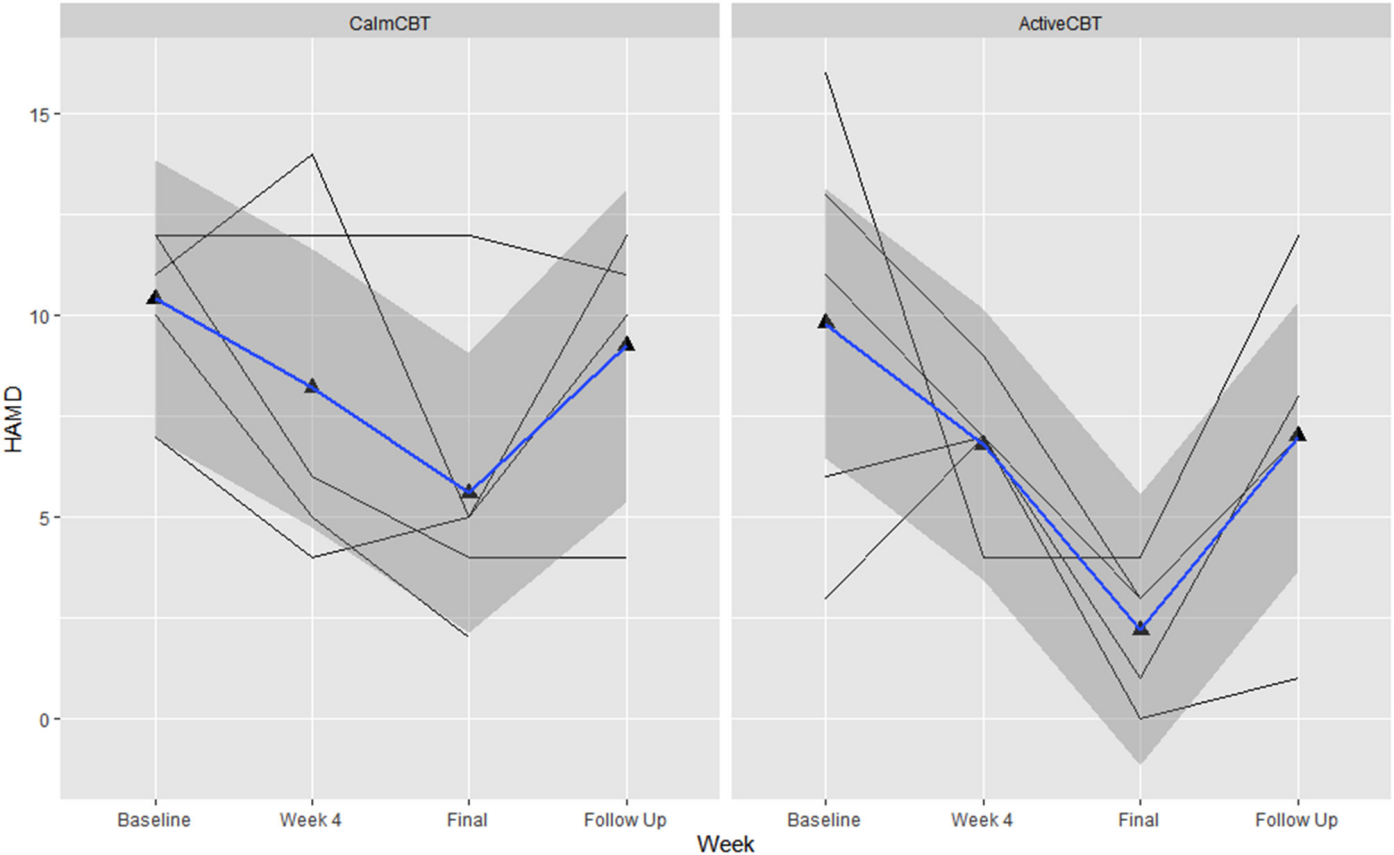
Changes in depression symptom severity on the Hamiilton Rating Scale of Depression-17 (HAMD) across the intervention. Data are total scores of the interviewer-administered HAMD self-reported at baseline, the final visit, and 3-month follow-up. Higher values indicate greater symptom severity, with values over 10 indicated moderate to severe depressive symptoms. B, baseline; F, final assessment; FU, 3-month follow-up.

**Figure 8:**
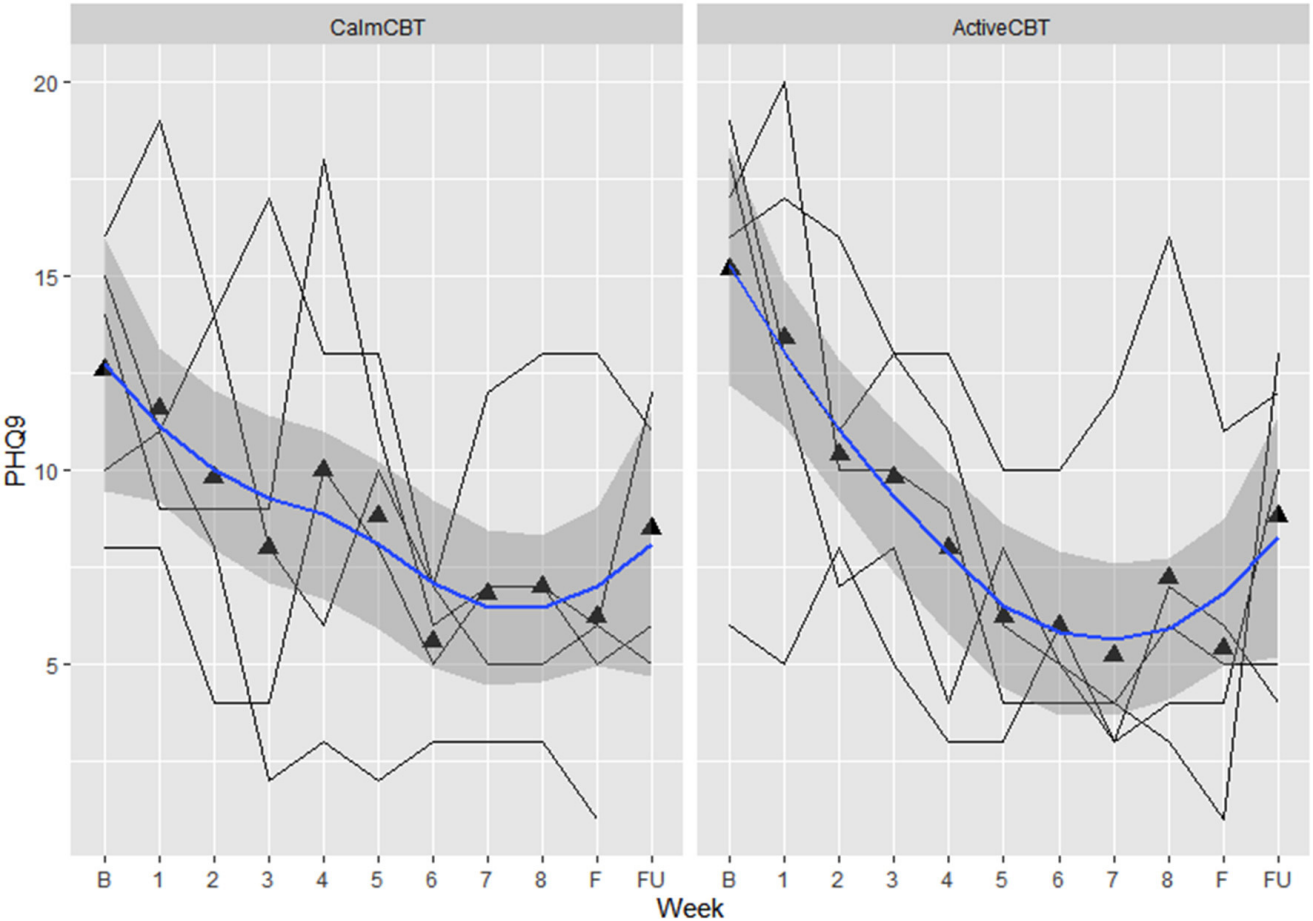
Changes in depression symptom severity on the Patient Health Questionnaire-9 across the intervention. Data are total scores of the PHQ-9 self-reported at baseline, all 8 therapy sessions (e.g., “1” indicating “CBT Session 1”), during the final visit, and 3-month follow-up. Higher values indicate greater symptom severity, with values over 10 indicated moderate to severe depressive symptoms. B, baseline; F, final assessment; FU, 3-month follow-up.

Depression outcomes in both groups were reduced immediately after the intervention, with a 60% remission rate (i.e., 3/5 participants) based on no longer meeting current criteria on the SCID for their initial depressive disorder in each group. At follow-up, remission rates were: 40% in Active (2/5 participants) and 25% in Calm (1/4 participants). Estimated means showed symptom severity (HAM-D Total) reductions favoring the ActiveCBT group from baseline to final (*g* = −1.33). At 3-month follow-up, estimated means again showed the ActiveCBT having a larger change though somewhat attenuated from final (*g* = −0.45). Similarly, estimated mean PHQ-9 scores decreases appeared larger from baseline to the final visit in the ActiveCBT group (*g* = −0.62). Symptoms in both groups remained improved at follow up, with a little difference between the groups (*g* = −0.19). Figure 7 shows depressive symptoms *via* HAMD and Figure 8 shows depressive symptoms on the PHQ-9 in both groups across time.

## Discussion

Overall, exercise priming in ActiveCBT to augment CBT for the treatment of DEP appears feasible, acceptable, and plausibly efficacious. Recruitment into the study was consistent and participants showed little trouble in scheduling and completing visits indicating high feasibility of the trial. High satisfaction of the treatment and adherence to the assigned conditions indicates the intervention had high acceptability. There were plausible effects both on proximally enhancing CBT within- and between-session effectiveness (e.g., greater behavioral activation: *g* = 1.40 difference in estimated mean between groups favoring ActiveCBT, working alliance: *g* = 1.10) and clinical outcomes of DEP (e.g., HAM-D reduction: *g* = −1.33, SCID: 60% remission in both groups at the final visit reduced to 40% in Active and 25% in Calm at 3-month follow-up). Overall, high feasibility, acceptability and potential efficacy for augmenting the antidepressant effects of CBT suggest exercise priming of CBT is worthy of further study.

A primary aim of this pilot study was to assess the feasibility of recruiting and retaining participants. The established partnerships for recruitment, steady influx of interested participants, and rapid progression of enrolled participants through the study demonstrate clear feasibility. Referral efforts from community partners were efficiently completed, and additional, available partnerships with larger neighboring cities were not needed for this pilot trial, though would likely be beneficial in future studies. Screening criteria also appear sufficient, though three participants who met inclusion criteria and were included in the study were not suffering from a current DEP episode (though did have a SCID-defined depressive disorder) and had lower symptom severity. Future research including participants with higher baseline levels of depressive symptoms could allow for a more fine-tuned analysis of changes in symptoms over time and between groups, so including, 1) participants in a current depressive episode (e.g., on SCID), and 2) who with higher current DEP symptom severity (e.g., HAM-D or PHQ-9 thresholds) as inclusion criteria may be helpful in future studies.

The present results also suggest high acceptability, as evident by excellent study and condition adherence (10/10 participants completed baseline, all intervention, and final sessions, with just one participant not completing the 3-month follow-up), high fidelity (7.45/10 average), and high overall participant satisfaction. Adherence to the overall intervention was above previously reported adherence to online CBT for DEP, as a meta-analysis found average rates of 84.5% and 61.5% for in-person and internet-based CBT session adherence, respectively (52). Adherence to the randomized pre-therapy condition (i.e., Active or Calm) was also monitored and differences in heart rate (107 median in ActiveCBT vs. 83 in CalmCBT) and step count (3,128 steps in ActiveCBT vs. 64 steps in CalmCBT) strongly indicate a successful experimental manipulation of pre-therapy activities. Notably, two participants chose non-walking modes of exercise that led to lower step counts and heart rates. The heart rate data from these commercial monitors indicate that participants may not have reached a moderate intensity and in-person research in future trials with enhanced exercise monitoring should lead to a clearer match with the prescribed moderate intensity. The specific intensity that each exercise was performed may not be critical for altering CBT mechanisms as past research indicates that mood benefits occur for depressed individuals after light exercise as moderate or hard intensity exercise (15). Still, given higher adherence to in-person CBT and greater internal validity with supervised exercise sessions, future research employing in-person study visits would be expected to reduce interindividual variability and may produce more robust results.

Therapy fidelity ratings were high (~75%), likely reflecting that the present study employed quality training methods, including an expert trainer who met individually with experienced coders to review the standardized manual and coding rubric, as recommended previously (53). Establishing inter-rater reliability post-training with subsequent checks would further improve fidelity in future trials. Interestingly, two sessions with lower fidelity ratings (i.e., 6/10) were perceived to be transformative (“breakthrough session”) by two participants, as they were specifically mentioned in the qualitative interviews, potentially indicating a necessary deviation from the standard therapy for participant growth. Additional measures of fidelity may be helpful for better understanding the quality of CBT implementation. Nonetheless, our acceptability measures (i.e., STSS and qualitative analysis) provide initial evidence of high-quality delivery, therapist-client rapport, and effectiveness of skills embedded in the standardized training. Taken together, this suggests sufficient training and supervision of the CBT and assessments, with moderate-to-high standardization and high patient satisfaction.

Intriguingly, preliminary efficacy data show that ActiveCBT may have led to large improvements in state anhedonia from before exercise (Pre) to between exercise and therapy (Mid) that did not appear to occur in those randomized to the Calm condition (between-group *g* = 1.44 from estimated means on the DARS), along with large differences in estimated mean working alliance across all 8 weeks (*g* = 1.10) and mean behavioral activation (*g* = 1.40), both favoring ActiveCBT. Differences in estimated level of behavioral activation and the frequency of automatic thoughts (*g* = −0.65), specifically, suggests exercise priming may augment key components of CBT to make it more effective (54–56). Moreover, a quicker development of therapeutic alliance in the Active condition could indicate a more general common factor benefit to exercise priming, as therapeutic alliance appears to be an important predictor of the success of not only CBT but also many other therapies (10, 56–59). Further, depressive symptom severity results showed large improvements in symptoms across the intervention (Figures 7, 8) with SCID remission of 60% in both groups. While the 3-month follow-up depression data indicate some increase in symptom severity from the final assessment, overall improvement from baseline was still apparent with remission on the SCID of 40% in Active and 25% in Calm. Given the potentially quicker development of therapeutic alliance and behavioral activation, along with a potential immediate and longer-term clinical benefit that appeared more pronounced in the Active group, exercise priming could be combined with different therapies and/or in other clinical populations to improve treatment effectiveness. However, more research that is appropriately powered will be needed to see if exercise priming is specifically able to enhance CBT's effects on short- and long-term clinical outcomes as well as how exercise priming may be applicable across conditions or other treatments.

This pilot trial has several limitations. Participants were actively recruited November 2020-March 2021, in which seasonal changes and the COVID-19 pandemic may have influenced mood and DEP. In the qualitative interviews, all participants reported that therapy was helpful and effective, though one participant (CalmCBT) did indicate changes in social interactions (e.g., visiting family over holidays) and weather that may have also contributed to symptom improvement. This study occurred during a global pandemic (i.e., COVID-19) that broadly resulted in worsening mental health (e.g., higher anxiety, depressive symptoms, stress) in US adults and restricted in-person study visits and therapy (60). Supervised exercise and in-person therapy sessions would allow greater internal validity to better identify potential mechanisms (i.e., state anhedonia, BDNF) of exercise priming, though our findings suggest the potential for generalizability in future trials (e.g., prescriptions of walking to therapy sessions, home exercise prior to tele-therapy). Finally, participant variability in primary diagnosis may have limited the ability to see differences in plausibility of efficacy between groups. While the effect sizes across multiple measures favoring the exercise priming group suggest this to be a potentially efficacious method for augmenting mechanisms of change in CBT and overall outcomes, the present small and homogenous sample with relatively mild DEP needs to be replicated in a larger sample to corroborate these promising initial results.

## Conculsions

Exercise priming of CBT sessions appears to be feasible, acceptable, and plausibly efficacious for enhancing mechanisms of CBT. Given the apparent reduction in anhedonia across exercise, and the appearance of quicker improvements in working alliance, and higher behavioral activation, exercise priming appears to be a promising augmentation strategy for improving DEP treatment. Future trials evaluating a population with Major Depressive Disorder and biological mechanisms are warranted.

## Data Availability Statement

The raw data supporting the conclusions of this article will be made available by the authors, without undue reservation.

## Ethics Statement

The studies involving human participants were reviewed and approved by Iowa State University Institutional Review Board. The patients/participants provided their written informed consent to participate in this study.

## Author Contributions

JM conceptualized the work and all authors assisted with the design and implementation of the project. JM, SP, JL, CB, and JS acquired and compiled the data and completed the qualitative analysis. ET and NW trained and supervised therapists and led fidelity assessments. TM assisted with data analysis, and D-cL guided exercise condition implementation and safety. All authors were involved in the interpretation of the data, drafting and revising the work critically for important intellectual content, approval of the final version of the manuscript and agree to be held accountable for all aspects of the work.

## Funding

This project was supported by an internal seed grant to JM from Iowa State University.

## Conflict of Interest

The authors declare that the research was conducted in the absence of any commercial or financial relationships that could be construed as a potential conflict of interest.

## Publisher's Note

All claims expressed in this article are solely those of the authors and do not necessarily represent those of their affiliated organizations, or those of the publisher, the editors and the reviewers. Any product that may be evaluated in this article, or claim that may be made by its manufacturer, is not guaranteed or endorsed by the publisher.
